# Efficacy of a Novel Bi-Steric mTORC1 Inhibitor in Models of B-Cell Acute Lymphoblastic Leukemia

**DOI:** 10.3389/fonc.2021.673213

**Published:** 2021-08-02

**Authors:** Bianca J. Lee, Sharmila Mallya, Nuntana Dinglasan, Amos Fung, Tram Nguyen, Lee-or Herzog, Joshua Thao, Edward G. Lorenzana, David Wildes, Mallika Singh, Jacqueline A. M. Smith, David A. Fruman

**Affiliations:** ^1^Department of Biology, Revolution Medicines, Inc., Redwood City, CA, United States; ^2^Department of Molecular Biology & Biochemistry, University of California, Irvine, CA, United States

**Keywords:** mTORC1, targeted (selective) treatment, 4EBP1, combination therapy, Ph+ B-ALL

## Abstract

The mechanistic target of rapamycin (mTOR) is a kinase whose activity is elevated in hematological malignancies. mTOR-complex-1 (mTORC1) phosphorylates numerous substrates to promote cell proliferation and survival. Eukaryotic initiation factor 4E (eIF4E)-binding proteins (4E-BPs) are mTORC1 substrates with an integral role in oncogenic protein translation. Current pharmacological approaches to inhibit mTORC1 activity and 4E-BP phosphorylation have drawbacks. Recently we described a series of bi-steric compounds that are potent and selective inhibitors of mTORC1, inhibiting 4E-BP phosphorylation at lower concentrations than mTOR kinase inhibitors (TOR-KIs). Here we report the activity of the mTORC1-selective bi-steric inhibitor, RMC-4627, in BCR-ABL-driven models of B-cell acute lymphoblastic leukemia (B-ALL). RMC-4627 exhibited potent and selective inhibition of 4E-BP1 phosphorylation in B-ALL cell lines without inhibiting mTOR-complex-2 (mTORC2) activity. RMC-4627 suppressed cell cycle progression, reduced survival, and enhanced dasatinib cytotoxicity. Compared to a TOR-KI compound, RMC-4627 was more potent, and its effects on cell viability were sustained after washout *in vitro*. Notably, a once-weekly, well tolerated dose reduced leukemic burden in a B-ALL xenograft model and enhanced the activity of dasatinib. These preclinical studies suggest that intermittent dosing of a bi-steric mTORC1-selective inhibitor has therapeutic potential as a component of leukemia regimens, and further study is warranted.

## Introduction

The target of rapamycin (TOR) is a serine/threonine kinase with an evolutionarily conserved function in nutrient sensing and cell growth (1, 2). In mammals, the mTOR protein functions in two complexes (mTORC1, containing raptor; mTORC2, containing rictor) with distinct regulation and substrates. mTORC1 integrates signals from nutrients and growth factors to promote cell growth and proliferation. mTORC2 is regulated by nutrient-independent mechanisms, and has a largely distinct set of substrates including AKT and other kinases of the AGC family. Both complexes have elevated activity in many tumors where they promote signaling and metabolic pathways that drive hallmarks of cancer (2, 3). Among mTORC1 substrates, the 4E-BP family (4E-BP1, 4E-BP2, 4E-BP3) are particularly important regulators of cell proliferation and transformation. When mTORC1 activity is low, hypophosphorylated 4E-BPs bind to eIF4E, displacing the scaffolding protein eIF4G within the cap-dependent translation initiation complex known as eIF4F. Activation of mTORC1 causes phosphorylation of 4E-BPs (on T37, T46 and S65 of 4E-BP1), leading to dissociation of 4E-BPs from eIF4E to allow eIF4F assembly and augment cap-dependent translation of a subset of cellular mRNAs important for tumorigenesis and maintenance of tumor growth (4, 5).

There is a strong conceptual basis for targeting the mTORC1/4E-BP axis in blood cancers. mTORC1 substrate phosphorylation is basally elevated in a variety of hematologic malignancies (3, 6). mTOR signaling has been particularly well documented in B-cell and T-cell acute lymphoblastic leukemias (B-ALL, T-ALL) (3, 6). These aggressive malignancies arise from lymphoid precursors and their expansion is driven by several factors including oncogenic mutations, epigenetic changes, and supportive signals from the microenvironment. In B-ALL patients, phosphorylated 4E-BP1 (p4E-BP1) correlates strongly with poor prognosis (7). In T-ALL cell lines and primary T-ALL and B-ALL samples, targeting of mTORC1 or eIF4F promotes apoptosis (8). Two generations of mTOR inhibitors have been studied extensively in acute leukemias and other blood cancers. Rapamycin and its analogs (rapalogs) are selective and potent mTORC1 inhibitors, active in the low nanomolar range to inhibit phosphorylation of a subset of mTORC1 substrates [e.g. S6 kinases (S6K)]. However, rapamycin is an allosteric inhibitor that minimally inhibits phosphorylation of T37 and T46 on 4E-BP1 (3, 9). In most blood cancer systems, rapamycin is weakly cytostatic and not cytotoxic. Second generation inhibitors, here termed mTOR kinase inhibitors (TOR-KIs, also known as pan-mTOR active-site inhibitors), were designed to be ATP-competitive and complete inhibitors of mTORC1 (3, 10, 11). In contrast to rapamycin, TOR-KIs strongly suppress 4E-BP phosphorylation and have improved anti-leukemic potential (12–16). However, they also inhibit mTORC2 activity, which might limit their therapeutic window. In addition, TOR-KIs are less potent than rapamycin, generally active in the mid-nanomolar range in cells, and lose cytotoxic activity after compound washout *in vitro* or metabolism *in vivo* (17).

Third-generation bi-steric mTORC1 inhibitors couple the high selectivity of rapamycin for mTORC1 with the broad mTOR kinase inhibitory effects of ATP-competitive TOR-KIs through covalent linkage of the two pharmacophores. The prototype inhibitor, RapaLink-1, achieves complete inhibition of mTORC1 with modest selectivity (approximately four-fold) over mTORC2 (18). We recently reported a series of novel mTORC1-selective bi-steric compounds generated by independently tuning the affinities of the rapamycin core and ATP mimetic moieties to modulate mTORC1/2 selectivity while maintaining potency for inhibition of 4E-BP1 phosphorylation (19). These compounds potently suppress phosphorylation of both S6K and 4E-BP1 at concentrations that spare inhibition of mTORC2 and phosphorylation of its substrate AKT on S473 *in vitro*. Following *in vivo* administration, mTORC1-selective bi-steric inhibitors cause tumor growth inhibition in xenograft models of breast cancer at tolerated doses.

The bi-steric mTORC1-selective inhibitor RMC-5552 is currently in clinical development. RMC-4627, which consists of a rapamycin monomer covalently linked to the TOR-KI PP242, is a bi-steric compound previously used in other preclinical studies (19). Using RMC-4627, we sought to test the anti-leukemic effects of selective and complete inhibition of mTORC1. We focused on Philadelphia Chromosome-positive (Ph+) B-ALL, a poor prognosis subtype driven by the *BCR-ABL* fusion oncogene that encodes a deregulated ABL tyrosine kinase. In *in vitro* cell line models of Ph+ B-ALL, RMC-4627 potently and selectively inhibited mTORC1, induced cell cycle arrest and apoptosis, and sustained inhibitory effects on signaling and cell viability following compound washout. Once weekly intraperitoneal administration of RMC-4627 as a single agent caused a dose-dependent reduction of leukemic burden in a xenograft model of human Ph+ B-ALL and enhanced the efficacy of the BCR-ABL tyrosine kinase inhibitor (TKI) dasatinib with acceptable *in vivo* tolerability. These preclinical findings demonstrate that intermittent dosing with a potent and selective mTORC1 inhibitor has therapeutic potential in hematologic malignancies and may be combined effectively with dasatinib. Adding a bi-steric mTORC1 inhibitor to therapeutic regimens could address the medical need to improve TKI responses in Ph+ B-ALL and other blood cancers driven by constitutively active tyrosine kinase signaling.

## Materials and Methods

### Inhibitors

The following inhibitors were purchased: MLN0128 (Active Biochem and Selleck Chemicals), MK-2206 (Active Biochem), dasatinib and rapamycin (LC Laboratories). Synthesis of RMC-4627 (alternatively known as BiS-13x) was previously described (19). All compounds were dissolved in DMSO at 10 mM.

### Cell Lines and Cell Culture Conditions

SUP-B15 cells (ATCC) were cultured in RPMI 1640 medium with 10% fetal bovine serum, 2 mM L-Glutamine, 10 mM HEPES, and 1% Penicillin/Streptomycin; or with additional supplementation (20% FBS and 0.05 mM Beta-mercaptoethanol). Cells were maintained in a 37°C humidified 5% CO_2_ incubator. p190 BCR-ABL transformed cells were generated as previously described (12).

### Inhibitor Washout Experiments

Cells were exposed to inhibitors or DMSO for 4 hours. Cells in the washout group were washed with PBS and plated in fresh media for the indicated additional time after washout.

### Immunoblot Analysis

Immunoblot analysis was performed as previously described (20). See **Supplementary Methods** for additional information.

### Phospho-Protein MSD Assay

mTOR substrate phosphorylation was assayed by MesoScale Discovery Multi-Array Assay Systems (MSD). Cells were exposed to serial 3-fold dilutions of compounds in complete medium for 2 hours. MSD was performed according to the manufacturers’ protocols. See **Supplementary Methods** for additional information.

### Cap-Binding Assay

Analysis of the cap-binding complex was performed by adapting a previously described protocol (21). See **Supplementary Methods** for additional information.

### Cell Cycle and Cell Death Analysis

Cells were fixed in 50% ethanol, washed, and stained in 0.1 mg/ml propidium iodide (PI)-staining solution (Life Technologies). Cells were analyzed on a FACSCalibur flow cytometer (Becton-Dickinson, San Jose, CA), and cell cycle analysis performed using FlowJo software v.5.7.2 (TreeStar).

After excluding cell debris, induction of cell death was measured by calculating the percentage of intact cells with Sub-G1 DNA content. Cell death was also measured using Annexin V (Life Technologies) and PI (Life Technologies) staining.

### Mice

Balb/c mice and NOD-*scid* IL2Rgamma^null^ (NSG) mice (Jackson Laboratory) were housed in specific pathogen-free conditions in barrier facilities. All procedures related to animal handling, care and treatment were performed according to the guidelines by the Institutional Animal Care and Use Committee (IACUC) following the guidance of the Association for Assessment and Accreditation of Laboratory Animal Care (AAALAC).

### SUP-B15 Xenograft Studies

See **Supplementary Methods** for details. Briefly, female NSG mice were implanted with SUP-B15 cells at 5-7 weeks of age. A small cohort was used for initial engraftment and cell expansion. Secondary transplants of larger cohorts were used for efficacy studies. In the monotherapy study, RMC-4627 was administered by intraperitoneal (ip) injections, once weekly (qw). In the combination study, RMC-4627 was administered ip qw, and dasatinib administered by oral gavage (po), once daily (qd).

### Flow Cytometry

See **Supplementary Methods** for details. Briefly, leukemic burden in SUP-B15 xenografts was monitored by staining live bone marrow cells with antibodies for surface markers (hCD19 and mCD45) or intracellular phospho-proteins (phospho-tyrosine, p4EBP1, pS6). Cells were analyzed on a CytoFLEX (Beckman Coulter), and data were analyzed using FlowJo software.

### Statistical Analysis

Statistical tests and some *P* values are reported in the Figure Legends. *P* values for Figure 2A and 4B are reported in **Supplementary Tables 1** and **2**, respectively. Symbols in the figure displays correspond to: ns (not significant), *P* > 0.05; *P ≤ 0.05; **P ≤ 0.01; ***P ≤ 0.001; **** *P* ≤ 0.0001.

## Results

### The mTORC1-Selective Bi-Steric Inhibitor, RMC-4627, Potently Suppresses mTORC1 Kinase Activity in B-ALL Cells

In this study, we used both human (SUP-B15) and mouse (p190 BCR-ABL-transformed bone marrow cells) B-ALL cell line models. SUP-B15 is a human Ph+ B-ALL cell line in which dasatinib strongly suppresses phosphorylation of BCR-ABL substrates and reduces proliferation in a concentration-dependent manner (12, 13). The term “p190 cells” refers to pools of murine pre-B leukemia cells established by infection of bone marrow lymphoid progenitors with a retroviral vector carrying a cDNA encoding the 190 form of BCR-ABL (12, 13). After 2-3 weeks in culture, these pre-B leukemia cells are nearly 100% positive for an IRES-linked marker (either eGFP or tail-less human CD4). In both SUP-B15 cells and p190 cells, high concentrations of dasatinib can block proliferation and survival; lower concentrations have partial effects that can be enhanced by co-treatment with mTOR inhibitors.

**Figure 1:**
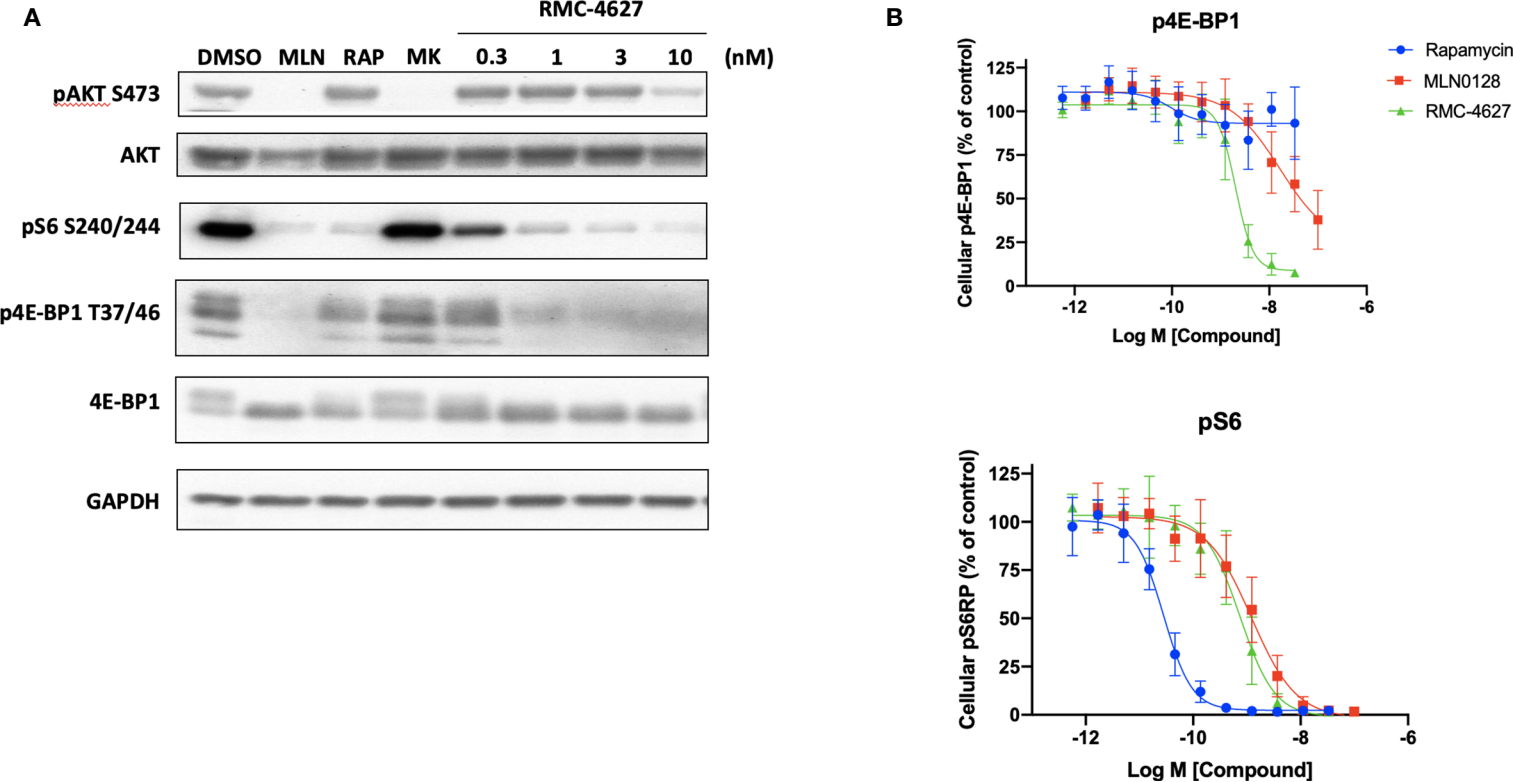
RMC-4627 selectively inhibits phosphorylation of mTORC1 substrates in human B-ALL cells. **(A)** Immunoblot analysis of cell lysates from SUP-B15 cells incubated with the indicated compounds for 2 hours (MLN: MLN0128, 100 nM; RAP: rapamycin, 10 nM; MK: MK2206, 100 nM). Similar results were observed in two additional experiments. **(B)** Levels of p4E-BP1 T37/T46 and pS6 S240/S244 determined by MesoScaleDiscovery (MSD) for cell lysates from SUP-B15 cells incubated with the indicated compounds for 2 hours. Data are expressed as mean percent of MSD signal (3 biological replicates) normalized to vehicle control, with error bars representing SD.

We previously demonstrated that the bi-steric mTORC1 inhibitor RMC-4627 potently inhibits phosphorylation of the mTORC1 substrates 4E-BP1 and S6K at concentrations that do not suppress phosphorylation of the mTORC2 substrate AKT S473, exhibiting 13- to 18-fold selectivity for mTORC1 in breast cancer cell lines *in vitro* (19). Here we carried out immunoblot experiments to compare mTOR substrate phosphorylation in SUP-B15 cells treated with rapamycin (10 nM), MLN0128 (100 nM), or increasing concentrations of RMC-4627. As expected, rapamycin strongly suppressed phosphorylation of the indirect mTORC1 substrate S6 protein (pS6 S240/S244) downstream of S6K, had a weak effect on p4E-BP1 T37/T46, and did not inhibit phosphorylation of the mTORC2 substrate AKT S473 (**Figure 1A**). In contrast, the TOR-KI compound MLN0128 inhibited phosphorylation of both mTORC1 and mTORC2 substrates. RMC-4627 exhibited a distinct pattern, with potent but selective inhibition of mTORC1 substrate phosphorylation. Specifically, pS6 was reduced upon treatment with concentrations as low as 0.3 nM; p4E-BP1 was reduced by 1 nM; and pAKT only partially at 10 nM RMC-4627 (**Figure 1A**). Similar results were observed in p190 cells (**Figure S1**). Using homogeneous, plate-based electrochemiluminescent assays to estimate potencies for inhibition of 4E-BP1 and S6 phosphorylation, we determined that RMC-4627 (p4E-BP1 EC_50_ = 2.0 nM; pS6 EC_50_ = 0.74 nM) was approximately 8- and 2-fold more potent than MLN0128 (p4E-BP1 EC_50_ = 16.5 nM; pS6 EC_50_ = 1.2 nM) in inhibiting p4E-BP1 and pS6, respectively, in SUP-B15 cells (**Figure 1B**).

### An mTORC1-Selective Inhibitor Induces Cell Cycle Arrest and Apoptosis in B-ALL Cells

**Figure 2:**
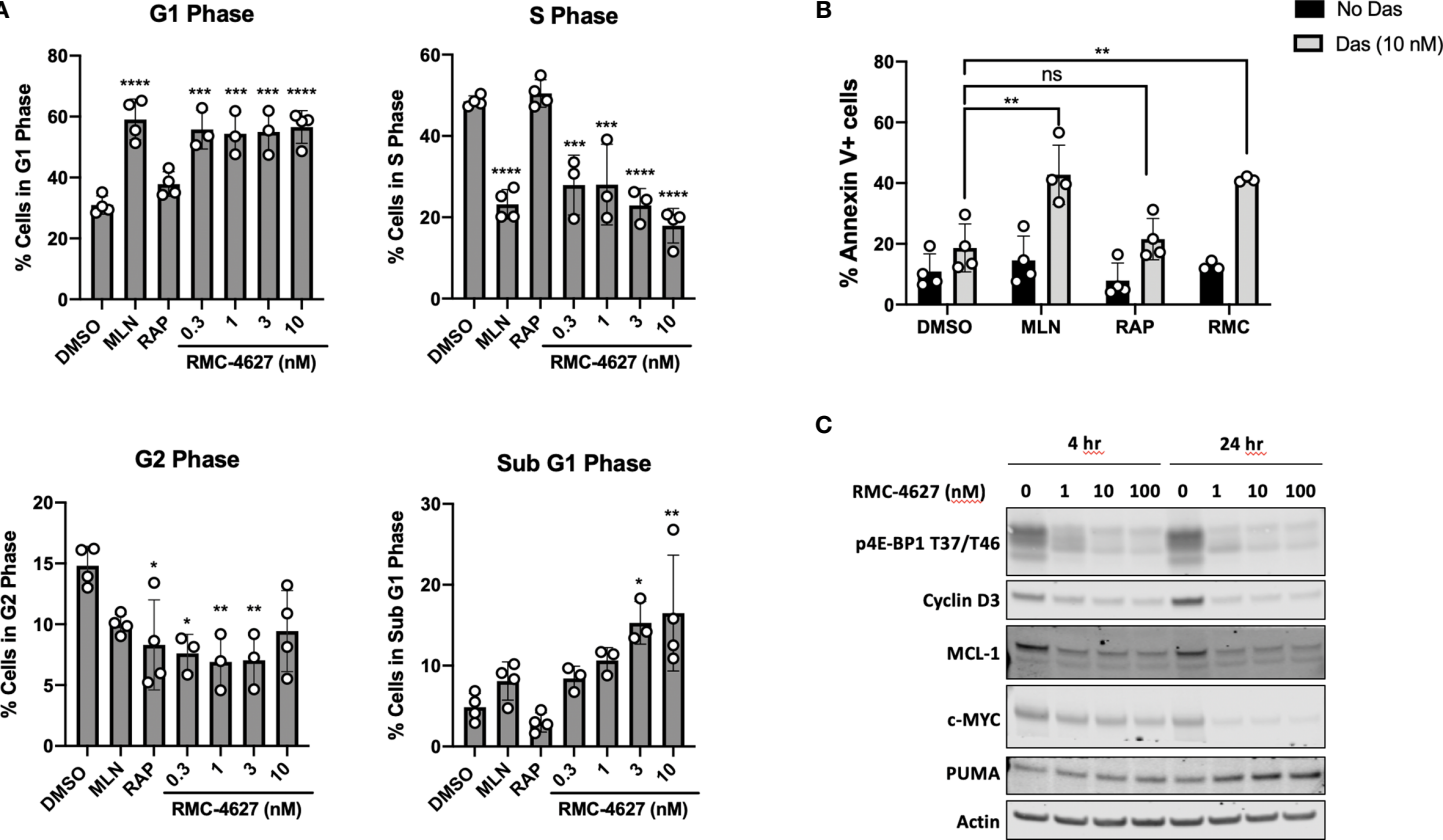
RMC-4627 decreases viability of human B-ALL cells as a single agent and in combination with dasatinib. **(A)** Propidium iodide staining and cell cycle analysis of SUP-B15 cells following incubation with the indicated compounds for 48 hours (MLN: MLN0128, 100 nM; RAP: rapamycin, 10 nM). Data are expressed as percent of cells in each phase, and are the mean of n=4 experiments with error bars representing SD. Statistical test: ordinary one-way analysis of variance (ANOVA) with post-hoc Tukey’s multiple comparison test (See **Supplementary Table 1** for exact *P* values and corresponding *, **, ***, and **** annotations). **(B)** Annexin V staining of SUP-B15 cells following incubation with MLN (MLN0128, 100 nM), RAP (rapamycin, 10 nM), and RMC-4627 (1 nM) in the absence or presence of dasatinib (10 nM) for 48 hours. Data are the mean of n=3 or 4 experiments with error bars representing SD. Statistical test: ordinary one-way analysis of variance (ANOVA) with post-hoc Tukey’s multiple comparison test (**P = 0.0011 for DMSO+Das and MLN+Das; ns *P* = 1.0 for DMSO+Das and RAP+Das; **P = 0.0053 for DMSO+Das and RMC+Das). **(C)** Immunoblot analysis of cell lysates from SUP-B15 cells starved for 24 hours in 0.1% FBS, then incubated with the indicated concentrations of RMC-4627 for 4 and 24 hours in the presence of 10% FBS. Data are a representative of three experiments with similar results.

The 4E-BP/eIF4E axis is a key mediator of cell cycle progression signals downstream of mTORC1 (22). In accord, and consistent with previous studies (12), the TOR-KI compound MLN0128 but not rapamycin caused a G1 cell cycle arrest in SUP-B15 cells (**Figure 2A**) and in p190 cells (**Figure S2A**). The mTORC1-selective inhibitor RMC-4627, at concentrations as low as 0.3 nM, induced cell cycle arrest to a similar degree as MLN0128 at a concentration of 100 nM (**Figures 2A** and **S2A**). Next, we measured apoptosis using two different assays. First, analysis of the cell cycle DNA content data (sub G1 phase) revealed low basal levels of apoptosis (~5% sub-diploid) in SUP-B15 and p190 cells that was increased slightly by MLN0128 but not rapamycin (**Figures 2A** and **S2A**). RMC-4627 caused a concentration-dependent increase in sub-diploid cells, with concentrations of 1 nM or greater increasing apoptosis to a similar or greater extent than 100 nM MLN0128 (**Figures 2A** and **S2A**). Second, we used Annexin-V staining and flow cytometry to measure directly the percentage of apoptotic cells after 48hr of compound exposure. Consistent with the DNA content studies, RMC-4627 caused a concentration-dependent increase in cell death (**Figures S2C** and **S3**). In addition, MLN0128 and RMC-4627 increased cell death in combination with a sub-maximal concentration of dasatinib in SUP-B15 and p190 cells (**Figures 2B** and **S2B**).

To investigate the mechanism of mTORC1-selective inhibition on cell cycle and survival, we measured expression of proteins that are particularly sensitive to changes in cap-dependent translation efficiency of their respective mRNAs. We took a candidate approach by assessing key regulators of cell cycle (cyclin D3) and apoptosis (MCL-1) (20). These experiments showed that in SUP-B15 cells RMC-4627 reduced levels of both proteins as early as 4hr after treatment with greater effects after 24hr (**Figures 2C** and **S4**). mTORC1 signaling in leukemia cells regulates other growth and survival factors besides cyclin D3 and MCL-1; for example, knockdown or inhibition of mTORC1 in human T-ALL cells reduces expression of the oncoprotein c-MYC while increasing expression of the pro-apoptotic protein PUMA (8). RMC-4267 caused similar changes to c-MYC and PUMA in SUP-B15 human Ph+ B-ALL cells (**Figures 2C** and **S4**).

### The Effects of an mTORC1-Selective Inhibitor on Signaling and Survival in B-ALL Cells Are Sustained After Washout

**Figure 3:**
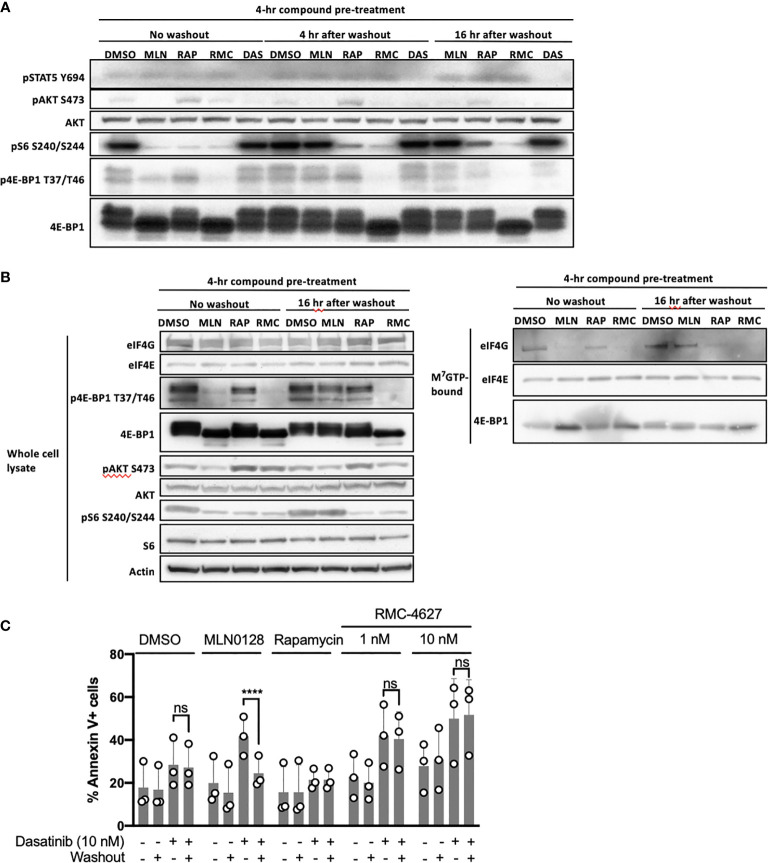
RMC-4627 exhibits sustained duration of action alone or in combination with dasatinib after washout. **(A)** Immunoblot analysis of cell lysates from SUP-B15 cells exposed to the indicated compounds for 4 hours followed by compound washout, and lysed 0, 4, and 16 hours after washout (MLN: MLN0128, 100 nM; RAP: rapamycin, 10 nM; RMC: RMC-4627, 3 nM; DAS: dasatinib, 10 nM). Data are a representative of three experiments with similar results. **(B)** Immunoblot analysis of whole cell lysates (left) and m^7^GTP pulldown assay (right) from SUP-B15 cells exposed to the indicated compounds for 4 hours followed by compound washout, and lysed 0 and 16 hours after compound washout (MLN: MLN0128, 100 nM; RAP: rapamycin, 10 nM; RMC: RMC-4627, 3 nM). Similar results were observed in a replicate experiment. **(C)** Annexin V staining of SUP-B15 cells exposed to the indicated compounds as single agents or in combination with dasatinib (10 nM) for 4 hours followed by compound washout, and analyzed by FACS 44 hours after washout (MLN0128, 100 nM; rapamycin, 10 nM). Data are the mean of n=3 experiments with error bars representing SD. Statistical test: two-way analysis of variance (ANOVA) with post-hoc Tukey’s multiple comparison test (ns *P*=0.98 for DMSO/Das+/Washout- and DMSO/Das+/Washout+; ****P < 0.0001 for MLN/Das+/Washout- and MLN/Das+/Washout+; ns *P* = 1.0 for RAP/Das+/Washout- and RAP/Das+/Washout+; ns *P* = 0.94 for RMC 1 nM/Das+/Washout- and RMC 1 nM/Das+/Washout+; ns *P* = 0.93 for RMC 10 nM/Das+/Washout- and RMC 10 nM/Das+/Washout+).

The data described above show that the mTORC1-selective inhibitor RMC-4627 is more potent on a molar basis than MLN0128, consistent with studies of earlier generation bi-steric inhibitors (such as RapaLink-1) compared to TOR-KIs (18). Another advantage of bi-steric mTORC1 inhibitors is their prolonged duration of action, which could be attributed to binding of the rapamycin moiety to abundant cellular FKBP12 (17, 18), or to the increased avidity of a dual binding site inhibitor compared to single binding site inhibitors. Sustained target inhibition, even after compound washout, offers the potential for intermittent dosing *in vivo* (17). We first tested the effect of RMC-4627 on mTORC1 inhibition in SUP-B15 cells *in vitro*, 4hr or 16hr after compound washout. RMC-4627 (10 nM) exhibited significant inhibition of mTORC1 phosphoprotein readouts (pS6 and p4E-BP1) for at least 16hr after compound washout (**Figure 3A**). Consistent with previous observations (17), the effect of rapamycin on pS6 was largely sustained, whereas the effects of MLN0128 on pS6 and p4E-BP1 were fully reversed within 4hr of washout (**Figure 3A**). Next, we used a cap-binding assay in which eIF4E and associated proteins are pulled down from cell lysates using beads coupled to m^7^GTP (20). Immunoblot of the total cell lysates from this experiment (**Figure 3B**, left) confirmed the effects of mTOR inhibitor washout on duration of substrate phosphorylation. Here, assembly of the active eIF4F cap-binding complex was assessed by co-association of the scaffolding protein eIF4G. Immunoblot of the cap-bound fraction (**Figure 3B**, right) showed that RMC-4627 reduced eIF4G association and increased 4E-BP1 association after 4hr of treatment, and this effect was sustained 16hr after washout. In contrast, the effect of MLN0128 on eIF4F assembly was largely reversed after washout. These biochemical effects correlated with distinct activity in viability assays. Specifically, the ability of mTOR inhibitors to enhance dasatinib cytotoxicity was sustained after washout for RMC-4627, but not for MLN0128 (**Figure 3C**).

### Anti-Leukemic Tumor Burden and Pharmacodynamic Activity of an mTORC1-Selective Inhibitor in a B-ALL Xenograft Model

**Figure 4:**
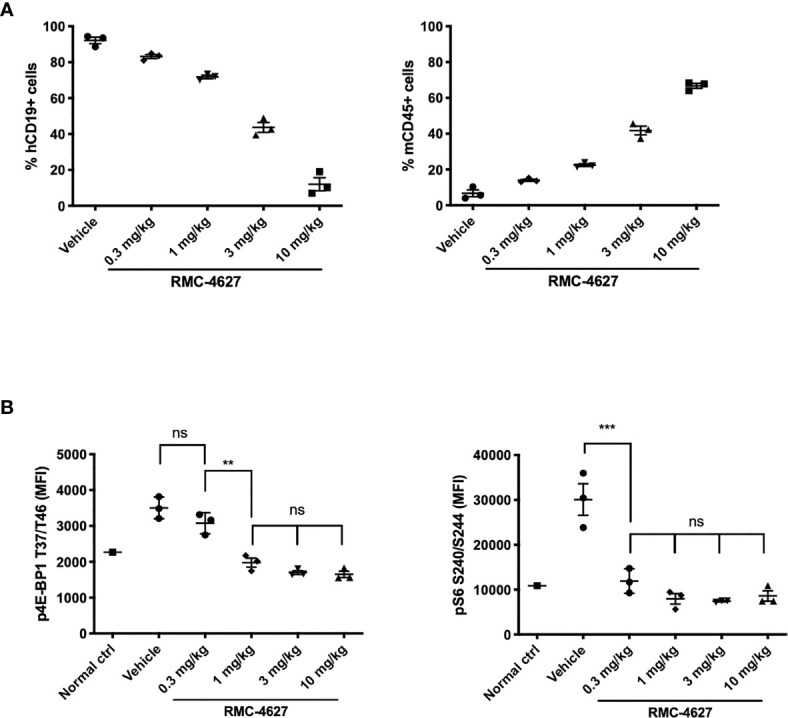
RMC-4627 reduces leukemic burden and suppresses phosphorylation of 4E-BP1 and S6 in a Ph+ B-ALL xenograft model. **(A)** Percentage of human leukemia (% hCD19+ cells) (left) and percentage of mouse lymphocytes (% mCD45+ cells) in bone marrow of SUP-B15 xenografts dosed with RMC-4627 (ip qw) for 4 weeks. Data are the mean percentages of each group (n=3; center line) with error bars representing SEM. **(B)** Levels of p4EBP1 T37/T46 and pS6 S240/S244 determined in bone marrow of SUP-B15 xenografts (live cell population) 4-5 hours after the final dose of a repeat dosing schedule with RMC-4627, and compared to a healthy age-matched control NSG mouse (normal ctrl). Data are the mean signal of each group (n=3; center line) with error bars representing SD. Statistical test: ordinary one-way analysis of variance (ANOVA) with post-hoc Tukey’s multiple comparison test (See **Supplementary Table 2** for exact *P* values and corresponding ns, **, and *** annotations).

We employed a Ph+ B-ALL xenograft model in which human SUP-B15 cells were injected intravenously into immunodeficient (NSG) mice. In this model, human CD19+ leukemia cells (hCD19+) are detectable in the bone marrow within 18-20 days and mice develop fatal disease after approximately 40 days. First, we conducted a dose titration of RMC-4627 monotherapy (0.3, 1, 3, and 10 mg/kg intraperitoneal), with once weekly (qw) treatments on days 19, 26, 33 and 40. Flow cytometric analysis of bone marrow cells demonstrated a dose-dependent reduction in leukemia burden (% hCD19+ cells) and a concurrent increase in the fraction of murine hematopoietic cells (mCD45+) (**Figures 4A** and **S5A**). The 3 mg/kg weekly dose reduced leukemic burden by more than 50% and the 10 mg/kg dose by nearly 90%. Pharmacodynamic measurements showed that RMC-4627 produced a dose-dependent reduction in both p4E-BP1 and pS6 in the leukemia cells, reaching levels approximating those in bone marrow cells from an age-matched healthy control mouse (**Figure 4B**). A dose of 0.3 mg/kg was sufficient to suppress pS6 nearly completely and normalize the spleen weights (**Figure S5B**), but had minimal effect on p4E-BP1 and % hCD19+ cells in the bone marrow. Reduced bone marrow leukemic burden was only achieved using doses of 1 mg/kg or higher, doses which all caused p4E-BP1 suppression to levels comparable to that of the normal control mouse (**Figure 4B**).

**Figure 5:**
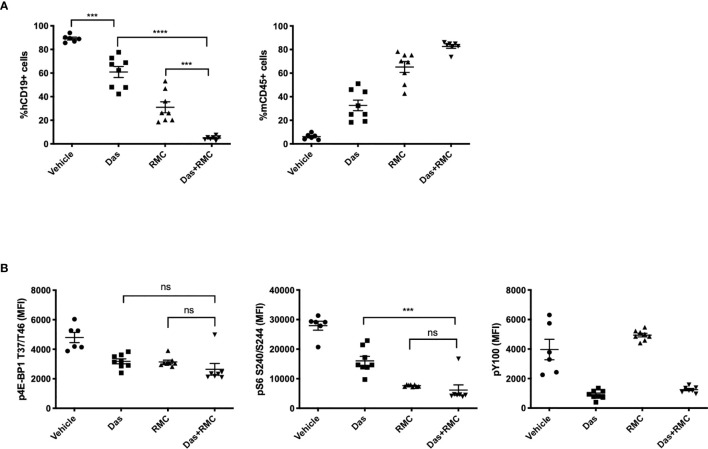
RMC-4627 enhances the anti-leukemic activity of dasatinib in a Ph+ B-ALL xenograft model. **(A)** Percentage of human leukemia (% hCD19+ cells) (left) and percentage of mouse lymphocytes (% mCD45+ cells) in bone marrow of SUP-B15 xenografts dosed with Vehicle, Das (dasatinib, 5 mg/kg, po qd), RMC (RMC-4627, 3 mg/kg, ip qw), and Das + RMC (dasatinib, 5 mg/kg, po qd; RMC-4627, 3 mg/kg, ip qw). In total, animals received 4 doses of RMC-4627 and 22 doses of dasatinib. Data are the mean percentages of each group (n = 8; center line) with error bars representing SEM. Statistical test: ordinary one-way analysis of variance (ANOVA) with post-hoc Tukey’s multiple comparison test (***P = 0.0001 for Vehicle and Das; ****P < 0.0001 for Das and Das+RMC; ***P = 0.0002 for RMC and Das+RMC). **(B)** Levels of p4E-BP1 T37/T46, pS6 S240/S244, and phosphotyrosine (pY100) determined in bone marrow of SUP-B15 xenografts (hCD19+ population) 4-5 hours after the final dose of the repeat dosing schedule (described above). Data are the mean signal of each group (n = 8; center line) with error bars representing SEM. Statistical test: ordinary one-way analysis of variance (ANOVA) with post-hoc Tukey’s multiple comparison test for p4E-BP1 (ns *P* = 0.46 for Das and Das+RMC; ns *P* = 0.53 for RMC and Das+RMC) and p-S6 (***P = 0.0001 for Das and Das+RMC; ns *P* = 0.86 for RMC and Das+RMC).

In order to model a translatable combination regimen, we chose a submaximal dose of RMC-4627 (3 mg/kg, qw, IP) for a combination study with a submaximal dose of dasatinib (5 mg/kg, qd, PO). The single and combined treatments were well tolerated overall and mice did not exhibit significant weight loss, although one animal from the combination group was euthanized nine days into treatment due to hypoactivity and dyspnea (**Figure S6A**). In the group of mice treated with dasatinib alone, there was a significant ~30% reduction in leukemia burden (**Figures 5A** and **S6B**). Treatment with RMC-4627 once weekly reduced leukemia burden by more than 50%, similar to the previous experiment. Notably, the combination treatment caused a near eradication of human leukemia cells from the bone marrow. Pharmacodynamic analysis confirmed that dasatinib strongly reduced tyrosine phosphorylation in leukemia cells, and caused partial suppression of mTORC1 phosphoprotein readouts (**Figure 5B**). As expected, RMC-4627 reduced mTORC1 substrate phosphorylation, but not phosphotyrosine levels. The combination of dasatinib with RMC-4627 inhibited mTORC1 substrate phosphorylation to an apparently greater extent than treatment with individual agents, but this did not reach statistical significance. These data show that a once weekly, well tolerated dose of RMC-4627 enhances the anti-leukemic effect of the tyrosine kinase inhibitor dasatinib in an aggressive *in vivo* model of human Ph+ B-ALL.

## Discussion

The observation of constitutive mTORC1 activity in aggressive B-cell tumors (3, 7) and mTOR inhibitor activity in preclinical models of B-cell leukemias has led to clinical trials assessing rapalogs and TOR-KIs in B-ALL (NCT00968253, NCT01184885, NCT02484430). However, these first and second-generation mTOR inhibitors have not yielded a clear therapeutic advantage at tolerated doses (23, 24). Key limitations of these studies are the lack of patient stratification or use of rationally designed combinations. Preclinical studies suggest that TOR-KIs might be particularly advantageous when combined with TKIs in malignancies driven by hyperactive tyrosine kinases (3). Although TK signaling is an important oncogenic driver in such tumors, signals from the microenvironment activate mTORC1 to sustain survival and leukemic expansion (14, 25). For example, our earlier preclinical studies of Ph+ B-ALL have shown that TOR-KIs (PP242, MLN0128) enhance the anti-leukemic activity of dasatinib (12, 13). In the Ph-like B-ALL subtype, MLN0128 enhances efficacy of dasatinib (in samples driven by ABL or PDGFR fusions) or ruxolitinib (in samples driven by active JAK signaling) (26, 27). Despite these promising findings, there remains concern that dual inhibition of mTORC1 and mTORC2 may be poorly tolerated in humans. Therefore, novel compounds are needed to address the continuing medical need for mTORC1 inhibition in therapeutic regimens for Ph+ B-ALL and other blood cancers.

In this study we have presented evidence that a third-generation, bi-steric mTORC1 inhibitor (RMC-4627) provides improved anti-leukemia activity compared to rapamycin and a TOR-KI compound in models of Ph+ B-ALL. RMC-4627 potently inhibits 4E-BP1 phosphorylation in B-ALL cells *in vitro* at concentrations approximately 8-fold lower than an investigational TOR-KI (MLN0128, also known as TAK-228 or sapanasertib). RMC-4627 at ~1 nM concentration had a similar effect as 100 nM MLN0128 on cell cycle and survival in human and mouse cell line models of Ph+ B-ALL. Moreover, RMC-4627 sustained inhibition of mTORC1 signaling and eIF4F assembly for at least 16hr after compound washout, and also enhanced the cytotoxic effect of dasatinib even after washout of the mTORC1 inhibitor. Previous work has studied the mechanism of prolonged action of bi-steric RapaLink compounds. Using FK506 competition, this work showed that stable binding to FKBP12 promotes cellular retention of bi-steric inhibitors and is required for sustained inhibition of mTORC1 after RapaLink washout (17, 18). This sustained effect translated into anti-tumor activity *in vivo*, in which once weekly dosing with RMC-4627 in a SUP-B15 xenograft model had single-agent activity, was well tolerated, and significantly enhanced the activity of dasatinib. Although we observed dose-dependent reduction of leukemic burden by RMC-4627 as a single agent, suppression of p4E-BP1 to the level of the normal control mouse was achieved by 1 mg/kg without further reduction observed at 3 or 10 mg/kg. Similarly, pS6 appeared to be fully inhibited at 0.3 mg/kg. In this study, phosphoprotein levels were measured at a single timepoint (4-5hr) following the final dose of RMC-4627 in a repeat dosing schedule. Thus, while low doses achieved full inhibition of substrate phosphorylation at this timepoint, higher doses may be required to achieve more sustained signaling inhibition that would translate to greater anti-tumor activity after repeat dosing. Alternatively, it is possible that the floor of the assays were reached with low doses of RMC-4627, thus precluding signal differentiation of higher doses. As a single agent, dasatinib also caused partial reduction in p4E-BP1 and pS6, which could be attributed to BCR-ABL-mediated signaling through the PI3K/mTOR pathway. The combination of RMC-4627 and dasatinib did not cause a statistically significant reduction in p4E-BP1 compared to either single agent or in pS6 compared to RMC-4627 alone. As discussed above, this could potentially be attributed to lower resolution at the floor of the assay. Furthermore, one animal in the combination group was particularly insensitive to p4E-BP1 and pS6 inhibition despite suppression of leukemic burden, thus affecting the mean phosphoprotein signal.

mTORC1 has numerous substrates that control expression or activity of key enzymes in metabolic and biosynthetic pathways necessary for tumor progression and maintenance (2, 3, 6). The 4E-BP/eIF4E axis is a key element of this broad regulatory program, controlling the cap-dependent translation of subsets of mRNAs important for oncogenic programs (4, 5). Here we tested two candidates whose mRNAs are highly sensitive to changes in cap-dependent translation efficiency: the cell cycle regulator cyclin D1 and the pro-survival protein MCL-1. RMC-4627 rapidly reduced the expression of both proteins. Consistent with a study of mTORC1 signaling outputs in T-ALL and B-ALL cells (8), RMC-4267 reduced expression of c-MYC and increased expression of PUMA. Further experiments are needed to identify the broader spectrum of translatome changes in leukemia cells treated with RMC-4627 and whether these and/or other mechanisms contribute to cell cycle arrest and apoptosis.

The limited clinical efficacy of TOR-KIs in early stage blood cancer trials (NCT02484430, NCT02752204) (24, 28) may be due in part to dose-limiting toxicities resulting from inhibition of mTORC2 and related kinases. The unique profiles of mTORC1-selective bi-steric inhibitors may provide a wider therapeutic index by maximizing mTORC1 target engagement at a level that was previously unachievable with TOR-KIs dosing regimens. We predict that the combination of high potency, high mTORC1 selectivity (19), and cellular retention (17) could enable these compounds to maintain continuous target coverage *in vivo* with reduced off-target activity and toxicity due to inhibition of other kinases. Thus, these preclinical findings suggest that an mTORC1-selective bi-steric inhibitor has the potential to provide an improved therapeutic window over TOR-KIs by achieving durable mTORC1 inhibition while minimizing inhibition of mTORC2 and related kinases, as well as provide the practical benefits of an intermittent dosing regimen, in hematologic malignancies.

B-ALL is treated with aggressive chemotherapy that is curative in the majority of pediatric cases but in a lower fraction of adolescent and adult patients. There are several high-risk subtypes that are refractory or develop resistance to chemotherapy, including Ph+ B-ALL. TKIs such as dasatinib are potent inhibitors of the BCR-ABL oncoprotein and have improved responses to chemotherapy in Ph+ B-ALL, yet many patients still relapse. In Ph-like B-ALL, studies are ongoing to assess the efficacy and tolerability of TKIs added to chemotherapy (NCT02420717, NCT03571321). Given the correlation of increased mTORC1 activity and high-risk in B-ALL (7), incorporating an mTORC1 inhibitor at an early stage in combination with TKIs offers the potential to increase complete responses and/or deepen remissions in preparation for stem cell transplant. Clinical management of high-risk B-ALL has improved due to introduction of TKIs, blinatumomab, CAR-T cell therapy and updated stem cell transplant protocols (29, 30). However, patients who are unable to go into remission, relapse on existing therapies, or are unable to tolerate immunotherapies or stem cell transplants still present an unmet medical need. Our preclinical studies suggest that combination of an mTORC1 inhibitor at a submaximal dose could lower the effective dose of a TKI and provide combinatorial benefit while potentially improving tolerability. Thus, introducing an effective mTORC1 inhibitor in combination with TKIs might further improve responses in Ph+ and Ph-like B-ALL, particularly in older adults who could benefit from long-term treatment with tolerable agents. Furthermore, many other hematologic malignancies display elevated mTORC1 signaling. These include T-ALL, non-Hodgkin’s lymphoma (NHL), and acute myeloid leukemia (AML) (31–35). As shown here through preclinical studies of combination with dasatinib in Ph+ B-ALL, pairing a bi-steric mTORC1 inhibitor with other precision therapies has the potential to achieve greater disease control at tolerated doses. Agents worth testing in combination studies include FLT3 inhibitors in AML, BCR signaling inhibitors in certain NHL subtypes, and BH3 mimetic drugs (BCL2 inhibitor venetoclax and MCL-1 inhibitors) in a variety of blood cancers. Our preclinical studies support the testing of bi-steric mTORC1 inhibitors in combinations that drive therapeutic benefit in hematological cancers.

## Data Availability Statement

The original contributions presented in the study are included in the article/**Supplementary Material**. Further inquiries can be directed to the corresponding authors.

## Ethics Statement

The animal study was reviewed and approved by Institutional Animal Care and Use Committee (IACUC) Association for Assessment and Accreditation of Laboratory Animal Care (AAALAC).

## Author Contributions

BL, SM, ND, AF, TN, LH, JT, EL, DW, and MS contributed to design, execution, and interpretation of experiments. DF and JS supervised and contributed to the design and interpretation of all experiments. DF and BL wrote the manuscript with significant input from JS, DW, and MS, and with contributions from all co-authors. All authors contributed to the article and approved the submitted version.

## Funding

LH was supported by T32 training grant AI060573 from the National Institutes of Health. DF has received research funding from Revolution Medicines.

## Conflict of Interest

DF has received research funding from Revolution Medicines. BL, ND, TN, EL, DW, MS, and JS are current or former employees of Revolution Medicines.

The remaining authors declare that the research was conducted in the absence of any commercial or financial relationships that could be construed as a potential conflict of interest.

## Publisher’s Note

All claims expressed in this article are solely those of the authors and do not necessarily represent those of their affiliated organizations, or those of the publisher, the editors and the reviewers. Any product that may be evaluated in this article, or claim that may be made by its manufacturer, is not guaranteed or endorsed by the publisher.
