# Supplementary material for: Efficacy of a Novel Bi-Steric mTORC1 Inhibitor in Models of B-Cell Acute Lymphoblastic Leukemia
Source: Front Oncol. 2021 Aug 2;11:673213. doi: 10.3389/fonc.2021.673213 (PMC8366290; doi:10.3389/fonc.2021.673213)
Supplement: Supplementary file 1 [file DataSheet_1.docx]

**Supplementary Methods**

*Immunoblot analysis*

The following primary antibodies were used: eIF4E, 4E-BP1, eIF4G, 4E-BP2, MCL-1, Cyclin D3, PUMA, phospho-4E-BP1 (T37/46), phospho-S6 (S240/244), phospho-AKT (S473), beta actin, GAPDH (Cell Signaling Technology, Beverly, MA); and c-MYC (Abcam, Burlingame, CA). Antibody dilutions were performed according to the manufacturer's instructions. Immunoreactive bands were visualized by enhanced chemiluminescence (GE Healthcare Life sciences, Pittsburgh, PA) after incubation with HRP conjugated secondary antibody (Promega, Madison, WI), or by an Odyssey Infrared Imaging System (LI-COR Biosciences, Lincoln, NE).

*Phospho-protein MSD assay*

mTOR substrate phosphorylation was assayed using the MesoScale Discovery Multi-Array Assay Systems for Phospho-4E-BP1 (Thr37/46) and Phospho-S6RP (Ser240/244) (MSD). Cells were exposed to serial 3-fold dilutions of compounds in complete medium with a final DMSO concentration of 0.1%. After 2 hours, cells were lysed, and MSD performed according to the manufacturers’ protocols. Samples were read using an MSD plate reader. Data were normalized to DMSO control values and plotted as a function of log M [compound]. Inhibitor EC_50_ was estimated using a sigmoidal concentration response (variable slope) model fitted to the data in Prism 7 (GraphPad Prism). In two of the replicate experiments, MSD signals were normalized to protein concentration prior to normalization to DMSO control values to adjust for variation in final lysate volume and concentration in some samples. Protein concentration was determined by bicinchoninic acid (BCA) protein assay (Thermo Scientific, Waltham, MA.

*Cap-binding assay*

Following incubation with inhibitors, 15-20x10^6^ cells were lysed by three freeze–thaw cycles in freeze–thaw lysis buffer. In all, 100–200 µg of protein from the lysates was then incubated with a suspension of 7-methyl-GTP-Agarose beads (Jena Bioscience, Germany), and placed on a rocker at room temperature. After 1 hour, the beads were pelleted and washed twice with lysis buffer. The beads were then boiled in running buffer for 5 minutes, separated by sodium dodecyl sulfate–polyacrylamide gel electrophoresis (SDS–PAGE), transferred to nitrocellulose membranes, and subjected to immunoblot analysis.

*SUP-B15 xenograft studies*

In all xenograft studies, female NSG mice were implanted with SUP-B15 cells at 5-7 weeks of age. A small cohort was used for initial engraftment and expansion of SUP-B15 cells, and secondary transplants of larger cohorts were used for efficacy studies. In the initial engraftment, animals were injected intravenously (iv) or retro-orbitally (ro) with 2x10^6^ SUP-B15 cells in 100 µl PBS. Progression of leukemic engraftment was monitored in periodically euthanized animals by determining percentage of human CD19 cells (hCD19+) in bone marrow by flow cytometry. 37 days after implantation, the remaining four animals were euthanized. Animals implanted by both routes exhibited similar engraftment (~70% hCD19+); thus, bone marrow from all animals were pooled and stored at -80°C. For both the monotherapy and combination efficacy studies, thawed bone marrow cells were transplanted by iv injection (0.25x10^6^ cells per animal), including additional animals (“monitor mice”) to determine leukemia progression. Engraftment was first assessed 18-19 days after transplant by collecting 100-500 μl of peripheral blood, isolating peripheral blood mononuclear cells (PBMCs) and determining % hCD19+ cells by flow cytometry. Upon detecting positive engraftment (>1% hCD19+ cells), mice were randomized into treatment groups, and dosing commenced 19-20 days after transplant. RMC-4627 was formulated in v/w/v, 5/5/90 Transcutol/Solutol HS 15/water, and dasatinib in v/v 50/50 Polypropylene Glycol/water. In the monotherapy study, RMC-4627 was administered by intraperitoneal (ip) injections, once weekly (qw). In the combination study, RMC-4627 was administered ip qw, and dasatinib administered by oral gavage (po), once daily (qd). In both studies, leukemic burden in the bone marrow (% hCD19+ cells) was measured weekly in monitor mice. 40 days after transplant and 4 hours after final dosing, treatment group animals were sacrificed, femurs were collected for bone marrow isolation (below), and spleen weights were measured.

*Flow cytometry*

Cell suspensions were obtained from peripheral blood collected via the tail vein, or from bone marrow cells isolated from the right and left femurs via centrifugation at 2000xg at 4°C for 10 minutes and flushing with PBS. Cells were incubated in Red Blood Cell Lysis Solution (Miltenyi Biotec, Germany) at room temperature in the dark for 7-10 minutes. After incubation, cells were washed twice with cold PBS, centrifuged at room temperature at 350xg for 5 minutes, and resuspended in FACs buffer (PBS + 2% FBS). Cell count and viability were determined using a Vi-CELL (Beckman Coulter, Brea, CA). To monitor leukemic progression in monitor mice throughout the study, live blood or bone marrow cells were stained for surface markers (hCD19 and mCD45) for flow cytometry analysis. At end of study, live bone marrow cells were first incubated in LIVE/DEAD^TM^ Fixable Violet Dead Cell Stain (Life Technologies, Carlsbad, CA) in the dark for 30 minutes, washed once with cold PBS, fixed with 2% PFA at room temperature for 15 minutes, and washed once with FACS buffer. A subset of each sample was also permeabilized in saponin for 10 minutes or in ice-cold 90% methanol overnight for intracellular phospho-protein staining.

Before staining, live or fixed cells were incubated with Fc block (1:50, Becton-Dickinson, San Jose, CA) for 10 minutes. For detection of leukemic burden by cell surface markers, cells were stained with the following antibodies (BioLegend, San Diego, CA) on ice for 15-30 minutes: PE-conjugated anti-human CD19 (1:100) and PerCP-Cy5.5-conjugated anti-mouse CD45 (1:100). For detection of intracellular phospho-proteins, cells were stained with the following antibodies (Cell Signaling Technologies, Danvers, MA): Alexa Fluor 647-conjugated anti-human Phospho-Tyrosine (P-Tyr-100)(1:100), or Alexa Fluor 488-conjugated anti-human Phospho-4E-BP1 (Thr37/46) (1:100), and Alexa Fluor 647-conjugated anti-human Phospho-S6 ribosomal protein (Ser240/244)(1:1000) at room temperature for 1 hour. Cells were washed and resuspended in FACS buffer, and analyzed on a CytoFLEX (Beckman Coulter, Brea, CA).

Data were analyzed using FlowJo software. Cell debris and doublet populations were excluded by forward and side scatter parameters and use of LIVE/DEAD^TM^ Fixable Violet Dead Cell Stain (Life Technologies, Carlsbad, CA). Cells were profiled for hCD19 and mCD45 surface marker expression. Leukemic burden of engrafted SUP-B15 cells was determined by % hCD19+/mCD45- cells. In the RMC-4627 monotherapy study, the mean fluorescence intensity (MFI) value for each intracellular phospho-protein was determined for the live cell population rather than the hCD19+ cell population. We were unable to detect hCD19 in these samples, which we attributed to cell permeabilization with methanol. In the subsequent combination study of RMC-4627 and dasatinib, cells were permeabilized with saponin, and MFI values for intracellular phospho-proteins were determined for the hCD19+ cell population.

**Supplementary Figure Legends**

**Supplementary Figure 1. RMC-4627 selectively inhibits phosphorylation of mTORC1 substrates in mouse B-ALL cells.**

Immunoblot analysis of cell lysates from p190 BCR-ABL-transformed mouse bone marrow cells (termed “p190 cells”) incubated with the indicated compounds for 2 hours (MLN: MLN0128, 100 nM; RAP: rapamycin, 10 nM; MK: MK2206, 100 nM). Similar results were observed in two additional experiments.

**Supplementary Figure 2. RMC-4627 decreases viability of mouse B-ALL cells as a single agent and in combination with dasatinib.**

**A**. Propidium iodide staining and cell cycle analysis of p190 cells following incubation with the indicated compounds for 48 hours (MLN: MLN0128, 100 nM; RAP: rapamycin, 10 nM). Data are expressed as percent of cells in each phase, and are the mean of n=2 experiments with error bars representing SD.

**B**. Annexin V staining of p190 cells following incubation with MLN0128 (MLN, 100 nM), rapamycin (RAP, 10 nM), and RMC-4627 (RMC, 1 nM) in the absence or presence of dasatinib (Das, 1 nM) for 48 hours. Data are the mean of n=3 experiments with errors bars representing SD. Statistical test: ordinary one-way analysis of variance (ANOVA) with post-hoc Tukey’s multiple comparison test (ns *P* = 0.057 for DMSO+Das and MLN+Das; ns *P* = 1.0 for DMSO+Das and RAP+Das; ***P* = 0.0012 for DMSO+Das and RMC+Das).

**C**. Annexin V staining of p190 cells following incubation with RMC-4627 at the indicated concentrations in the absence or presence of dasatinib (Das, 1 nM) for 48 hours. Data are the mean of n=2 experiments with errors bars representing SD.

**Supplementary Figure 3. RMC-4627 causes a concentration-dependent decrease in viability as a single agent and in combination with dasatinib in human B-ALL cells.**

Annexin V staining of SUP-B15 cells following incubation with RMC-4627 at the indicated concentrations in the absence or presence of dasatinib (Das, 1 nM) for 48 hours. Data are the mean of n=3 experiments with errors bars representing SD.

**Supplementary Figure 4. RMC-4627 alters protein levels of key regulators of cell cycle and survival in human B-ALL cells.**

Densitometry of immunoblot analysis from Figure 2C of cell lysates from SUP-B15 cells starved for 24 hours in 0.1% FBS, then incubated with the indicated concentrations of RMC-4627 for 4 and 24 hours in the presence of 10% FBS. Data are a representative of three experiments with similar results.

**Supplementary Figure 5. RMC-4627 reduces leukemic burden in a Ph+ B-ALL xenograft model.**

**A**. Flow cytometry analysis of bone marrow of one representative animal per group, with gates showing quantification of human leukemia cells (hCD19+) and mouse lymphocytes (mCD45+) in bone marrow of SUP-B15 xenografts dosed with RMC-4627 (ip qw) for 4 weeks (4 doses). The identity of the hCD19-/mCD45- population is unknown, but might represent leukemia cells that have downregulated expression of hCD19.

**B**. Mean spleen weights of n=3 animals following repeat dosing with RMC-4627 (ip qw) for 4 weeks (4 doses), and compared to a healthy age-matched control NSG mouse (normal ctrl). Error bars represent SEM.

**Supplementary Figure 6. RMC-4627 enhances the anti-leukemia activity of dasatinib and is well tolerated in a Ph+ B-ALL xenograft model.**

**A**. Mean percent body weight change of SUP-B15 xenografts dosed with Vehicle, Das (dasatinib, 5 mg/kg, po qd), RMC-4627 (3 mg/kg, ip qw), and Das + RMC-4627 (dasatinib, 5 mg/kg, po qd; RMC-4627, 3 mg/kg, ip qw), with error bars representing SD. In total, animals received 4 doses of RMC-4627 and 22 doses of dasatinib. One animal from the combination group was euthanized on day 9 due to hypoactivity and dyspnea; thus it was included in the mean percent body weight change only for days 0 and 7.

**B**. Flow cytometry analysis of bone marrow of one representative animal per group, with gates showing quantification of human leukemia cells (hCD19+) and mouse lymphocytes (mCD45+) in bone marrow of SUP-B15 xenografts on a repeat dosing schedule (described above). The identity of the hCD19-/mCD45- population is unknown, but might represent leukemia cells that have downregulated expression of hCD19.
