# Supplementary figures and images for: Efficacy of a Novel Bi-Steric mTORC1 Inhibitor in Models of B-Cell Acute Lymphoblastic Leukemia
Source: Front Oncol. 2021 Aug 2;11:673213. doi: 10.3389/fonc.2021.673213 (PMC8366290; doi:10.3389/fonc.2021.673213)

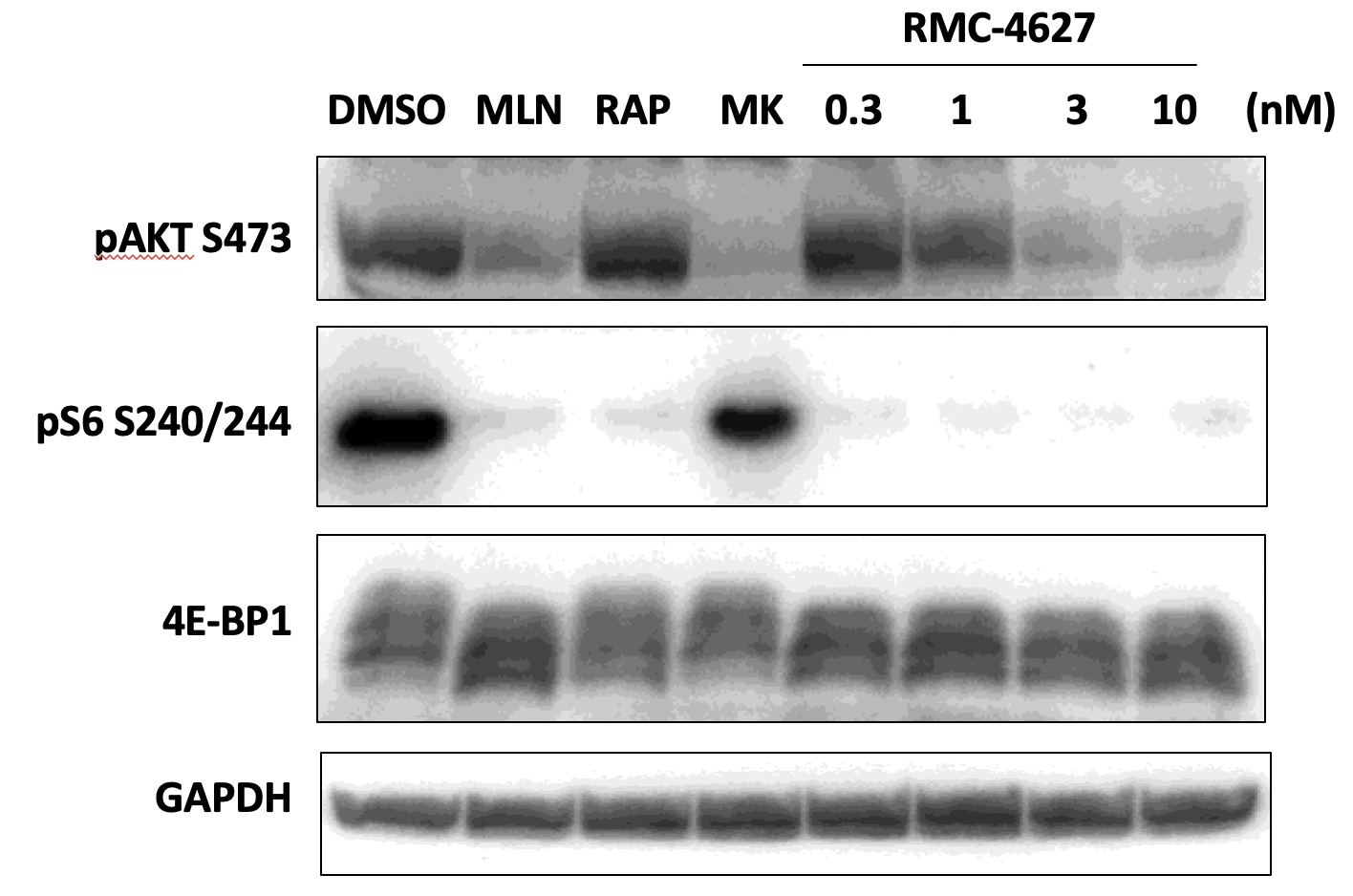

Supplement: Supplementary file 2 [file Image_1.tiff]

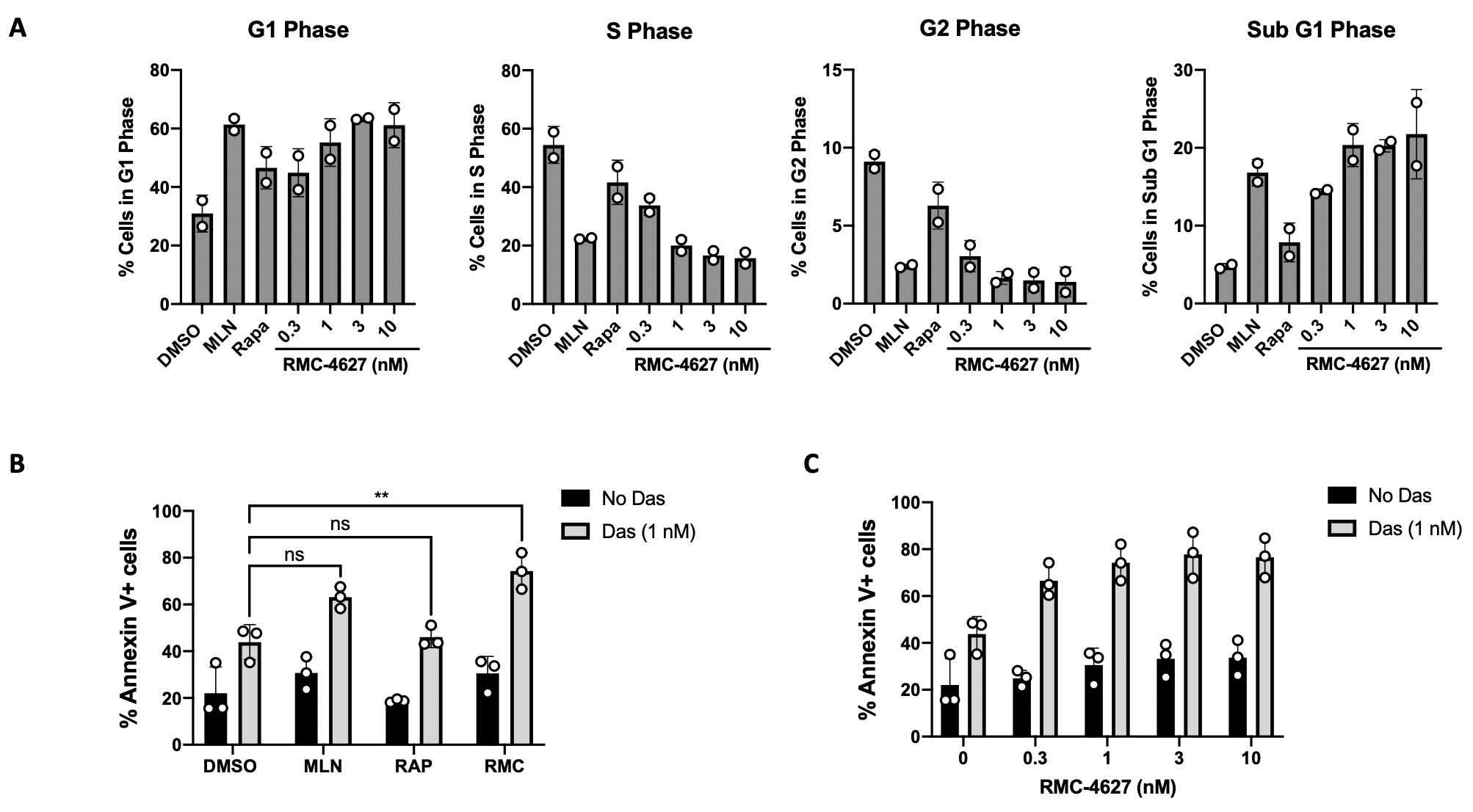

Supplement: Supplementary file 3 [file Image_2.tiff]

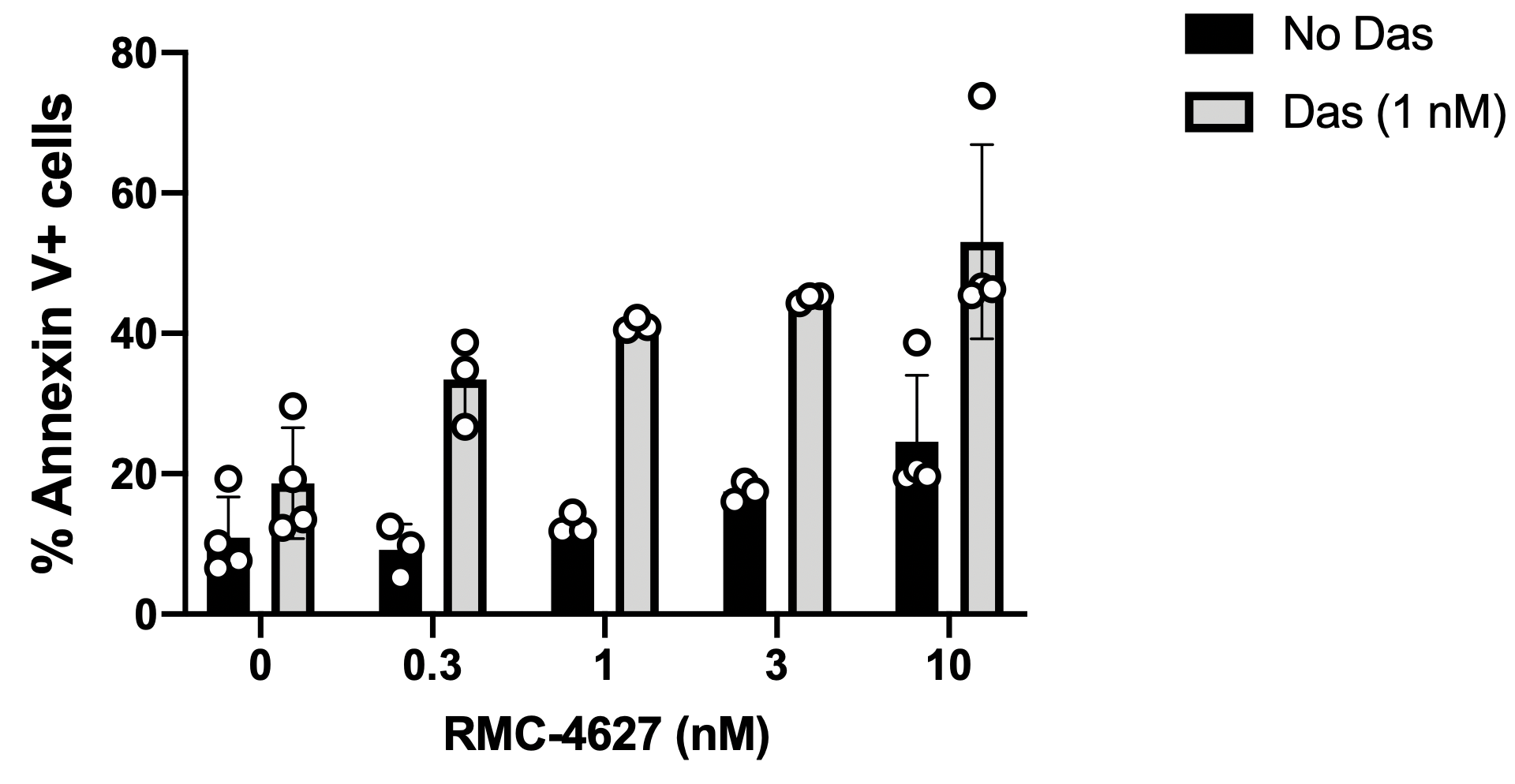

Supplement: Supplementary file 4 [file Image_3.tiff]

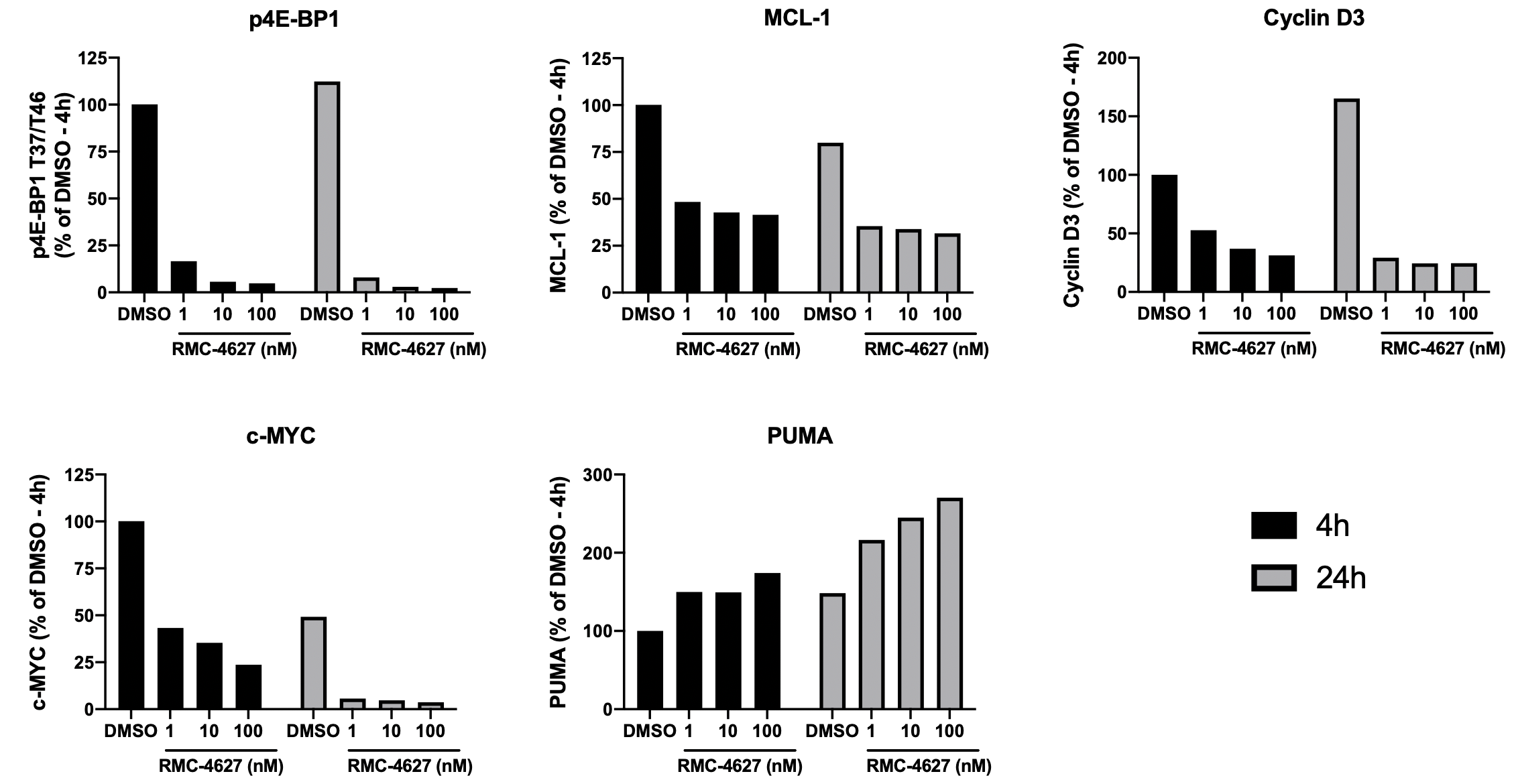

Supplement: Supplementary file 5 [file Image_4.tiff]

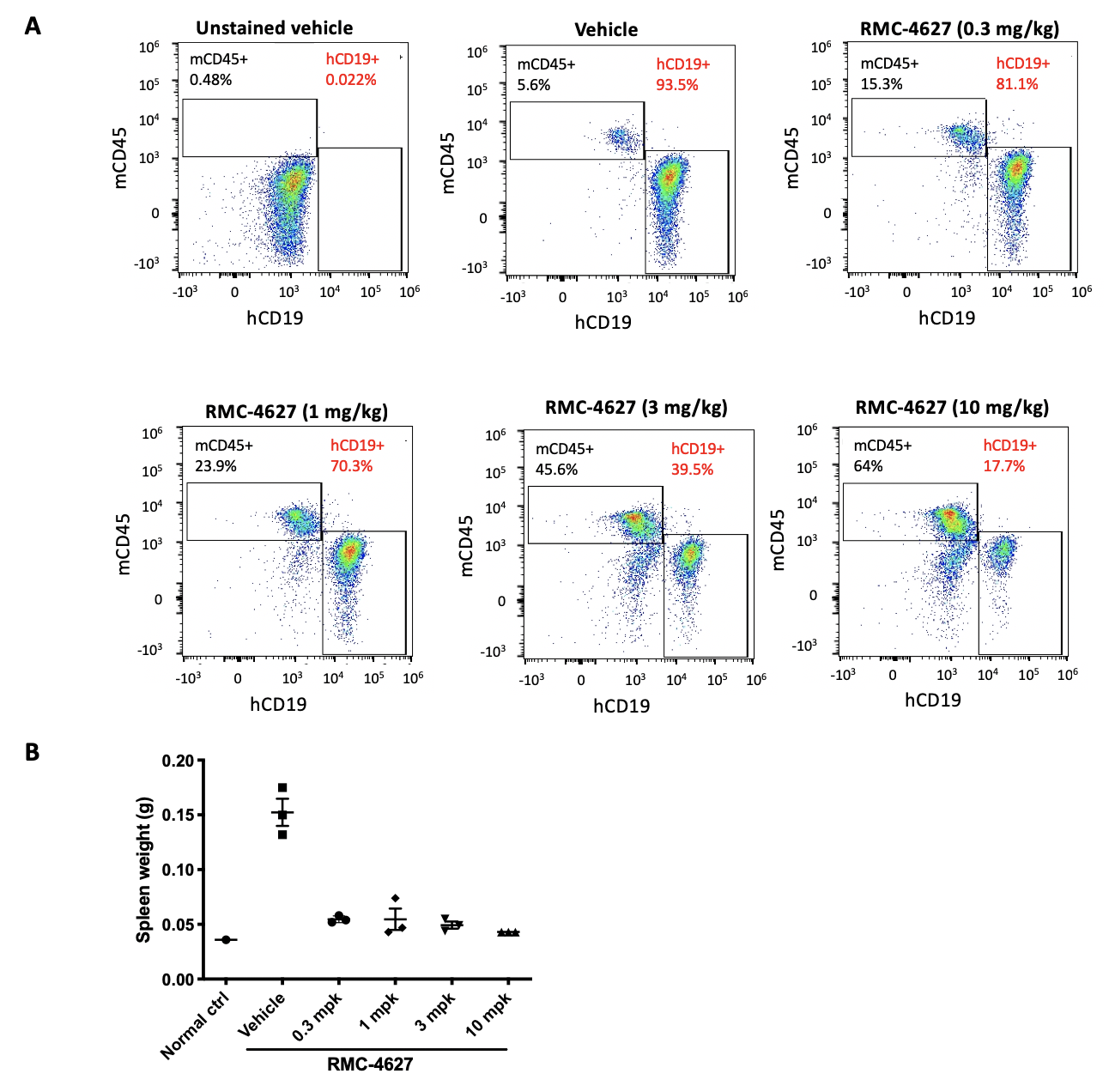

Supplement: Supplementary file 6 [file Image_5.tiff]

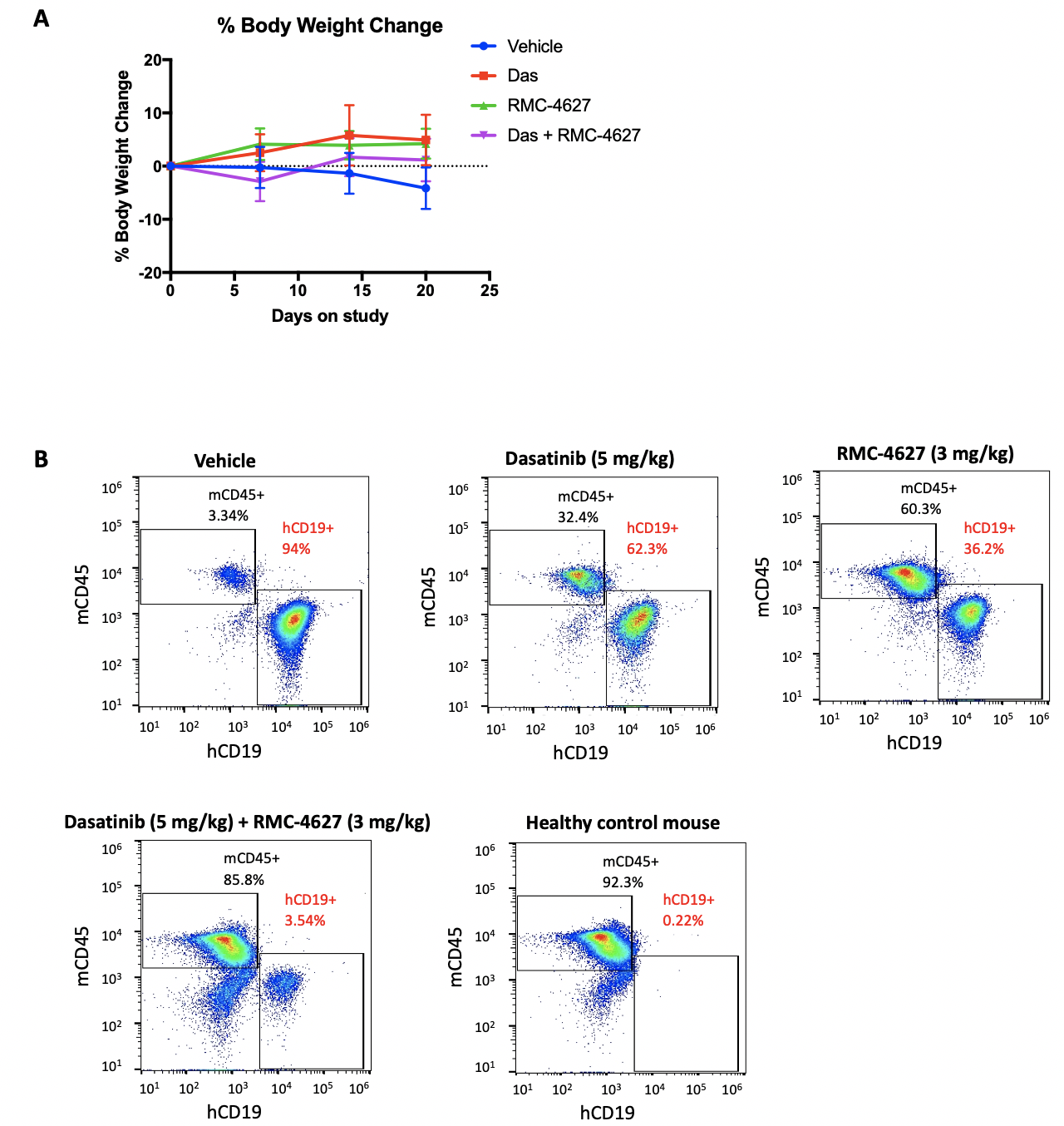

Supplement: Supplementary file 7 [file Image_6.tiff]
