# Supplementary material for: Efficacy of a Novel Bi-Steric mTORC1 Inhibitor in Models of B-Cell Acute Lymphoblastic Leukemia
Source: Front Oncol. 2021 Aug 2;11:673213. doi: 10.3389/fonc.2021.673213 (PMC8366290; doi:10.3389/fonc.2021.673213)
Supplement: Supplementary file 8 [file Table_1.pdf]

Supplementary Table 1: SUP-B15 cell cycle statistical analysis (for Figure 2A)

Statistical test: ordinary one-way analysis of variance (ANOVA) with post-hoc Tukey's test

**G1 Phase**	Summary	Adjusted P Value
DMSO vs. MLN	****	<0.0001
DMSO vs. RAP	ns	0.6155
DMSO vs. 0.3	***	0.0004
DMSO vs. 1	***	0.0007
DMSO vs. 3	***	0.0005
DMSO vs. 10	****	<0.0001
**S phase**	Summary	Adjusted P Value
DMSO vs. MLN	****	<0.0001
DMSO vs. RAP	ns	0.9981
DMSO vs. 0.3	***	0.0008
DMSO vs. 1	***	0.0009
DMSO vs. 3	****	<0.0001
DMSO vs. 10	****	<0.0001
**G2 phase**	Summary	Adjusted P Value
DMSO vs. MLN	ns	0.1397
DMSO vs. RAP	*	0.0215
DMSO vs. 0.3	*	0.0178
DMSO vs. 1	**	0.0082
DMSO vs. 3	**	0.0094
DMSO vs. 10	ns	0.0781
**Sub G1 phase**	Summary	Adjusted P Value
DMSO vs. MLN	ns	0.8205
DMSO vs. RAP	ns	0.9838
DMSO vs. 0.3	ns	0.8076
DMSO vs. 1	ns	0.3298
DMSO vs. 3	*	0.0114
DMSO vs. 10	**	0.002
