# Supplementary material for: Efficacy of a Novel Bi-Steric mTORC1 Inhibitor in Models of B-Cell Acute Lymphoblastic Leukemia
Source: Front Oncol. 2021 Aug 2;11:673213. doi: 10.3389/fonc.2021.673213 (PMC8366290; doi:10.3389/fonc.2021.673213)
Supplement: Supplementary file 9 [file Table_2.pdf]

Supplementary Table 2: SUP-B15 xenograft p4E-BP1 and pS6 statistical analysis (for Figure 4B)

Statistical test: ordinary one-way analysis of variance (ANOVA) with post-hoc Tukey's test

**p4E-BP1**	Summary	Adjusted P Value
Vehicle vs. 10 mg/kg	****	<0.0001
Vehicle vs. 3 mg/kg	****	<0.0001
Vehicle vs. 1 mg/kg	****	<0.0001
Vehicle vs. 0.3 mg/kg	ns	0.2741
Normal ctrl vs. 10 mg/kg	ns	0.2588
Normal ctrl vs. 3 mg/kg	ns	0.3328
Normal ctrl vs. 1 mg/kg	ns	0.8668
Normal ctrl vs. 0.3 mg/kg	ns	0.0925
10 mg/kg vs. 3 mg/kg	ns	0.9997
10 mg/kg vs. 1 mg/kg	ns	0.5296
10 mg/kg vs. 0.3 mg/kg	***	0.0002
3 mg/kg vs. 1 mg/kg	ns	0.6813
3 mg/kg vs. 0.3 mg/kg	***	0.0002
1 mg/kg vs. 0.3 mg/kg	**	0.0015
**pS6**	Summary	Adjusted P Value
Vehicle vs. Normal ctrl	**	0.0043
Vehicle vs. 10 mg/kg	***	0.0001
Vehicle vs. 3 mg/kg	****	<0.0001
Vehicle vs. 1 mg/kg	****	<0.0001
Vehicle vs. 0.3 mg/kg	***	0.0004
Normal ctrl vs. 10 mg/kg	ns	0.9875
Normal ctrl vs. 3 mg/kg	ns	0.9294
Normal ctrl vs. 1 mg/kg	ns	0.9635
Normal ctrl vs. 0.3 mg/kg	ns	0.9997
10 mg/kg vs. 3 mg/kg	ns	0.9969
10 mg/kg vs. 1 mg/kg	ns	0.9998
10 mg/kg vs. 0.3 mg/kg	ns	0.8039
3 mg/kg vs. 1 mg/kg	ns	>0.9999
3 mg/kg vs. 0.3 mg/kg	ns	0.5601
1 mg/kg vs. 0.3 mg/kg	ns	0.6732
